# Prevalence and impact of Thiopurine S-methyltransferase (TPMT) and Nudix hydrolase 15 (NUDT15) polymorphisms on the occurrence of myelosuppression in Thai patients with inflammatory bowel disease receiving thiopurine therapy

**DOI:** 10.1093/crocol/otag115

**Published:** 2026-10-09

**Authors:** Rujirada Phoojaroenchanachai, Thanaboon Chaemsupaphan, Onuma Sattayalertyanyong, Nichcha Subdee, Phutthaphorn Phaophu, Marianee Salaemae, Julajak Limsrivilai

**Affiliations:** Division of Gastroenterology, Department of Internal Medicine, Faculty of Medicine Siriraj Hospital, Mahidol University, Bangkok, Thailand; Division of Gastroenterology, Department of Internal Medicine, Faculty of Medicine Siriraj Hospital, Mahidol University, Bangkok, Thailand; Division of Gastroenterology, Department of Internal Medicine, Faculty of Medicine Siriraj Hospital, Mahidol University, Bangkok, Thailand; Division of Gastroenterology, Department of Internal Medicine, Faculty of Medicine Siriraj Hospital, Mahidol University, Bangkok, Thailand; Division of Gastroenterology, Department of Internal Medicine, Faculty of Medicine Siriraj Hospital, Mahidol University, Bangkok, Thailand; Division of Gastroenterology, Department of Internal Medicine, Faculty of Medicine Siriraj Hospital, Mahidol University, Bangkok, Thailand; Division of Gastroenterology, Department of Internal Medicine, Faculty of Medicine Siriraj Hospital, Mahidol University, Bangkok, Thailand

**Keywords:** inflammatory bowel diseases, thiopurine, myelosuppression, TPMT, NUDT15, polymorphisms

## Abstract

**Introduction:**

Thiopurine-induced myelosuppression (TIM) is an important side effect influenced by 2 main genetic polymorphisms, thiopurine-S-methyltransferase (TPMT) and NUDT15. The data on Southeast Asian inflammatory bowel disease (IBD) patients remain limited. Our study aimed to determine the relationship between TPMT and NUDT15 polymorphisms and TIM following thiopurine treatment in Thai patients with IBD.

**Methods:**

We performed a retrospective cohort study of IBD patients receiving azathioprine with active follow-up at the IBD clinic. TPMT and NUDT15 polymorphisms were checked. Medical records were reviewed for TIM occurrence and its genetic associations.

**Results:**

A total of 124 IBD patients (73 Crohn’s disease, 51 ulcerative colitis; mean age 46.9 years) were included. Initial azathioprine dosing was 0.87 mg/kg/day, titrated to 1.54 mg/kg/day. TPMT variants were found in 6 patients (4.8%; 5 heterozygous and 1 homozygous) and NUDT15 variants in 21 patients (16.9%; 20 heterozygous and 1 homozygous). Over a 7.6-year follow-up, TIM occurred in 12 patients (9.7%). TIM rate was not significantly different among TPMT polymorphisms (wild-type 9.3%, heterozygous 20%, homozygous 0%), but was significantly different among NUDT15 polymorphisms (7.8% wild-type and heterozygous/homozygous 19%). The cumulative rates of TIM at 6, 12, and 60 months were 2.1%, 3.2%, and 7.2% in wild type and 0%, 5%, and 10% in NUDT15 heterozygous mutation. The only participant with a homozygous mutation developed TIM within the first month.

**Conclusion:**

NUDT15 polymorphisms are more common than TPMT variants in this study and are significantly associated with TIM. Pre-treatment genotyping may be considered, taking into account local mutation prevalence, test availability, and cost-effectiveness.

## Graphical Abstract

**Graphical Abstract:**
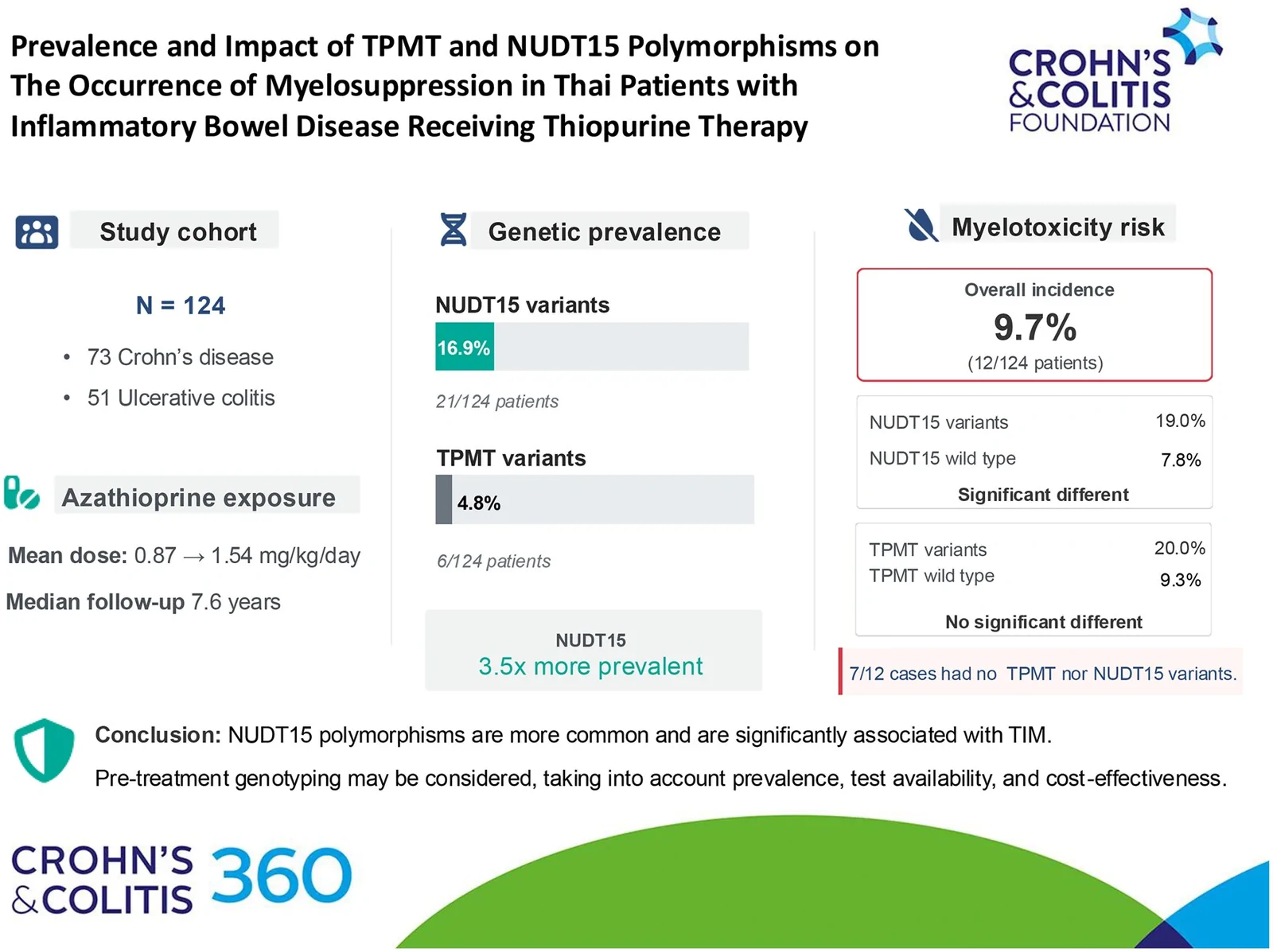

## Introduction

Inflammatory bowel diseases (IBD), consisting of ulcerative colitis (UC) and Crohn’s disease (CD), are chronic relapsing gastroenterological conditions that significantly impact long-term health and quality of life worldwide.^1^ Thiopurines, consisting of 6-mercaptopurine (6-MP), azathioprine (prodrug of 6-MP), and thioguanine, serve as cornerstone therapies for patients with moderate to severe disease activity or corticosteroid dependence.^2–4^ Furthermore, strong evidence supports the benefit of combination therapy of thiopurines with biological agents, mainly anti-tumor necrosis factor (anti-TNF), in improving clinical remission compared to monotherapy.^5^^,^^6^ Despite this evidence, side effects remain a significant factor influencing the clinician’s decision to initiate or discontinue thiopurines. The major adverse effects include an increased risk of infection, myelosuppression, hepatotoxicity, and certain malignancies.^7^^,^^8^ Thiopurine-induced myelosuppression (TIM) is a common and potentially life-threatening condition, with a cumulative incidence of 7% and an incidence of severe myelotoxicity of 1.1%.^8^ Active thiopurine metabolites are methylated by the thiopurine-S-methyltransferase (TPMT) enzyme into the inactive metabolite, 6-MMP. Genetic variants that reduce TPMT activity result in excessive cytotoxic metabolites, increasing the risk of severe TIM.^9^ Similarly, the nucleoside diphosphate-linked moiety X-type motif 15 (NUDT15) gene enhances diphosphatase activity, converting active thiopurine metabolites into inactive forms. Polymorphisms in the NUDT15 gene lead to impaired enzyme function, resulting in the accumulation of toxic metabolites (thioguanine triphosphate; TGTP) and an increased risk of myelotoxicity.^10^ These two genetic polymorphisms are the most common known risk factors associated with the development of TIM. The prevalence of variant TPMT genotypes is higher in Caucasians (10.1%) compared to South Asians (2.0%) and Chinese populations (4.7%).^11^ In contrast, the prevalence of NUDT15 variants is more common in Asia. A recent systematic review, primarily from East Asian countries, identified the heterozygous c.415C > T (C/T) diplotype as the most common NUDT15 variant, which is associated with leukopenia.^12^ Emerging evidence has therefore reinforced the role of preemptive NUDT15 genotyping before thiopurine therapy to predict and reduce the risk of thiopurine-induced myelotoxicity, particularly in Asian populations.^13^

While these genetic variants have been extensively studied in various populations, data in Southeast Asian countries remain limited. This knowledge gap underscores the need for research to better understand the prevalence and potential association of TPMT and NUDT15 polymorphisms on TIM in IBD patients, particularly in this region. The findings would help determine whether testing for these genetic variants prior to treatment initiation would be advantageous.

## Methods

### Study participants

This single-center, retrospective cohort study was conducted at the IBD clinic, Siriraj Hospital, Mahidol University, Thailand, from June 1, 2022, to August 31, 2023. We recruited all IBD patients who had been prescribed thiopurines and were actively followed up. Participants received standard treatment tailored to the severity of their diseases. Blood samples were collected from participants to analyze for TPMT and NUDT15 polymorphisms. Written informed consent was obtained, and a retrospective chart review was performed to identify TIM and associated clinical details. The study protocol was approved by the Siriraj Institutional Review Board on May 23, 2022 (approval number Si 399/2022).

### Data collection

Blood collection was obtained at study enrollment to determine major TPMT alleles that are associated with lower TPMT activity compared to wild allele (TPMT*1), including TPMT*2 (283G > C), TPMT*3A (460G > A, 719A > G), TPMT*3B (460G > A), TPMT*3C (719A > G), and TPMT*6 (539A > T). To identify TPMT*1, *2, *3A, *3B, and *3C variants, genotyping was performed using a multiplex Amplification Refractory Mutation System-Polymerase Chain Reaction (ARMS-PCR) method. For the detection of TPMT*6, the Polymerase Chain Reaction-Restriction Fragment Length Polymorphism (PCR-RFLP) technique was employed. All reagents for analyses were sourced from the PCRBIO HS Taq DNA Polymerase kit (PCRBIO, UK).^14^^,^^15^ NUDT15 c.415C > T C/T genotype (R139C variant) was detected from 200–300 ng of gDNA using specific primers at varying concentrations to simultaneously amplify wild-type and mutant alleles in PCR buffer (Qiagen, Hilder, Germany).^16^

Demographic data were retrospectively obtained from medical chart review, including age at diagnosis, disease classification and behavior, and laboratory investigations at thiopurine initiation. Doses of thiopurine initiated and titrated during the disease course were also collected. In our practice, complete blood counts and liver function tests were routinely monitored at every visit during thiopurine initiation, dose escalation, and maintenance treatment. Patients were usually assessed 1-2 months after thiopurine initiation or dose escalation, and every 3-6 months during maintenance treatment. Additional laboratory assessments were performed whenever clinically indicated. TIM was defined as the presence of myelosuppression after at least 7 days of thiopurine use, meeting 1 or more of the following criteria: white blood cell count < 3000 cells/mm^3^, neutrophil count < 1500 cells/mm^3^, or platelet count < 100 000 cells/mm^3^.^17^ The onset of TIM, along with laboratory findings and final outcomes, was also retrospectively ascertained through review of medical records.

### Statistical analysis

Categorical variables are reported as frequencies and percentages. Continuous data are expressed as the mean with standard deviation (SD) for normally distributed variables or as the median with the range or interquartile range (IQR) for non-normally distributed variables. Differences between variables were analyzed using the *t*-test for continuous data and the Chi-square test or Fisher’s exact test for categorical data, as appropriate. The Kaplan-Meier method was used to construct survival curves, and the log-rank test was applied to compare the effects of each genetic polymorphism on the TIM. A *P*-value of <.05 was considered statistically significant. All analyses were performed using SAS software (SAS Institute Inc., Cary, NC, USA).

## Results

### Baseline characteristics

Out of 498 patients at the Siriraj IBD clinic, 124 were prescribed thiopurine therapy, including 73 with CD and 51 with UC. The cohort comprised 54 males and 70 females with a mean age of 46.9 ± 16.7 years. Females were significantly more prevalent in the CD group (38.7% vs. 20.2%, *P *= .01), while there was no gender difference in UC (23.4% male vs. 17.7% female, *P *= .48). Baseline demographic data are shown in Table 1. Among CD participants, the ileocecum was the most common phenotype (27/73, 36.99%). Among UC participants, pancolitis was the most common presentation (29/51, 56.9%), followed by left-sided colitis (18/51, 35.3%).

**Table 1:** Baseline demographic data.

Parameter	All (*n* = 124)	Crohn’s disease (*n* = 73)	Ulcerative colitis (*n* = 51)
**Age (years)**	46.9 + 16.7	47.8 + 18.0	45.6 + 14.7
**Male (%)**	54 (43.5)	25 (20.2)	29 (23.4)
**Crohn’s disease**			
**Age at diagnosis (A1/A2/A3) (%)**		3 (4.11)/32 (43.84)/38 (52.05)	
**Location (L1/L2/L3/L4) (%)**		27 (36.99)/13 (17.81)/30 (41.10)/3 (4.11)	
**Behavior (B1/B2/B3/B3p) (%)**		47 (64.38)/11 (15.07)/7 (9.59)/8 (10.96)	
**Ulcerative colitis**			
**Disease extent (E1/E2/E3) (%)**			4 (7.84)/18 (35.29)/29 (56.86)
**Investigations prior to thiopurine initiation**			
**Hemoglobin (g/dL)**	11.96 + 1.93	11.89 + 1.81	12.05 + 2.11
**Mean corpuscular volume (fL)**	81.34 + 9.46	81.14 + 9.50	81.61 + 9.48
**White blood cell count (×10^9^/L)**	10.42 + 7.81	10.61 + 9.40	10.14 + 4.63
**Absolute neutrophil count (×10^9^/L)**	6.95 + 3.39	6.89 + 2.94	7.03 + 3.96
**Absolute lymphocyte count (×10^9^/L)**	2.02 + 0.92	1.98 + 1.05	2.08 + 0.72
**Platelet count (/mm^3^)**	394 857 + 138 484	406 239 + 149 734	378 021 + 121 312
**Aspartate aminotransferase (U/L)**	17.88 + 7.82	17.72 + 7.96	18.14 + 7.70
**Alanine aminotransferase (U/L)**	17.52 + 10.72	16.72 + 9.57	18.76 + 12.35

Data are shown as *N* (%) and mean ± SD, as appropriate.

All thiopurine prescriptions in the clinic were azathioprine. Dosage adjustments were made during regular visits by IBD physicians based on disease severity, adverse effects, and monitoring of complete blood counts and liver function tests in line with standard practice. Of all, the mean starting dose of azathioprine was 0.87 ± 0.23 mg/kg/day, with a mean final dose of 1.54 ± 0.51 mg/kg/day after titration. Eighty-nine patients, 49 (67.1%) with CD and 40 (78.4%) UC, could tolerate thiopurine treatment and underwent dose titration, with a median time to reach the maximum dosage of 12.8 months (range 3.9-35.3). Median time to achieve the maximum dose was not statistically different between CD and UC (10.5 months vs. 21.6 months, *P *= .16).

### Prevalence of TPMT and NUDT15 genotype

TPMT polymorphisms were identified in 6 out of 124 participants (4.8%), with 5 participants exhibiting the heterozygous TPMT*1/*3C phenotype and 1 participant exhibiting the homozygous TPMT *3B/*3C genotype. NUDT15 polymorphisms were detected in 21 participants (16.9%), comprising 20 heterozygous C/T and 1 homozygous T/T case. No participants had concurrent TPMT and NUDT15 polymorphisms.

### Thiopurines induced myelosuppression during therapy influenced by TPMT and NUDT15 polymorphisms

Over a median follow-up period of 7.6 years (IQR 4.3–17.6), TIM occurred in 12 out of 124 participants (9.68%). The median time to TIM after reaching the maximum thiopurine dosage was 12.0 months (IQR 0.5–35.7). Table 2 details the characteristics of TIM and the time to onset after thiopurine initiation. Among the 6 participants with TPMT polymorphisms, only 1 (16.7%) developed TIM, associated with the *1/*3C genotype, while the remaining TIM cases occurred in patients with wild-type TPMT. Overall, the occurrence rate of TIM was 9.3% (11/118) in participants with wild-type TPMT, 20% (1/5) in those with a heterozygous, and 0% (0/1) in the participant with a homozygous polymorphism. There was no significant difference in TIM rates among the TPMT polymorphism groups (*P *= .73).

**Table 2:** Characteristics of thiopurine-induced myelosuppression and associated genetic polymorphisms.

Patients	Disease	TPMT	NUDT15	Dose at TIM (mg/day)	Onset of TIM (months)	Hb (g/dL)	MCV (fL)	WBC (/mm^3^)	ANC (/mm^3^)	Platelet (/mm^3^)
**1/F**	CD	*1/*1	C/C	100	8.4	9.2	92.7	2390	1200	230 000
**2/F**	UC	*1/*1	**C/T**	50	26.8	11.4	94.9	2580	1150	229 000
**3/M**	UC	*1/*1	**T/T**	50	1.2	9.8	80	1440	28	380 000
**4/M**	CD	*1/*1	**C/T**	125	66.7	11.5	80	2720	1500	169 000
**5/F**	CD	*1/*1	C/C	75	24.7	11.4	90.2	2040	1040	121 000
**6/M**	CD	*1/*1	**C/T**	50	0.9	10.2	80.5	2680	710	227 000
**7/F**	UC	*1/*1	C/C	50	17.9	11.8	85.2	2980	680	300 000
**8/M**	CD	*1/*1	C/C	150	25.2	7	87.8	2310	1250	199 000
**9/M**	UC	*1/*1	C/C	50	85.5	15.9	80.1	2740	1930	229 000
**10/F**	CD	*3C/*1	C/C	75	47.6	12.2	100	2780	1480	272 000
**11/M**	UC	*1/*1	C/C	100	2.1	8.1	77.3	1630	1340	198 000
**12/M**	CD	*1/*1	C/C	50	3.0	6.8	83	3000	1780	505 000

Abbreviations: ANC: absolute neutrophil counts, CD: Crohn’s disease, F: female, Hb: hemoglobin, M: male, MCV: mean corpuscular volume, NUDT15: nucleoside diphosphate-linked moiety X motif 15, TIM: thiopurine-induced myelosuppression, TPMT: thiopurine-S-methyltransferase, UC: ulcerative colitis, WBC: white blood cell count.Genetic polymorphisms are indicated in bold.

Among 21 participants with NUDT15 polymorphisms, 4 developed TIM: 3 with heterozygous and 1 with homozygous polymorphisms. The occurrence rate of TIM was 7.8% (8/103) in those with wild-type NUDT15 and 19.0% (4/21) in heterozygous carriers or homozygous mutation. The cumulative TIM rates at 6, 12, and 60 months were 2.1%, 3.2%, and 7.2% in wild-type NUDT15, 0%, 5%, and 10% in heterozygous carriers. The only participant with a homozygous mutation developed TIM early, within the first month. The overall difference among groups was statistically significant (*P *< .0001, Figure 1). Notably, 7 of 12 participants who developed TIM had neither TPMT nor NUDT15 polymorphisms.

**Figure 1:**
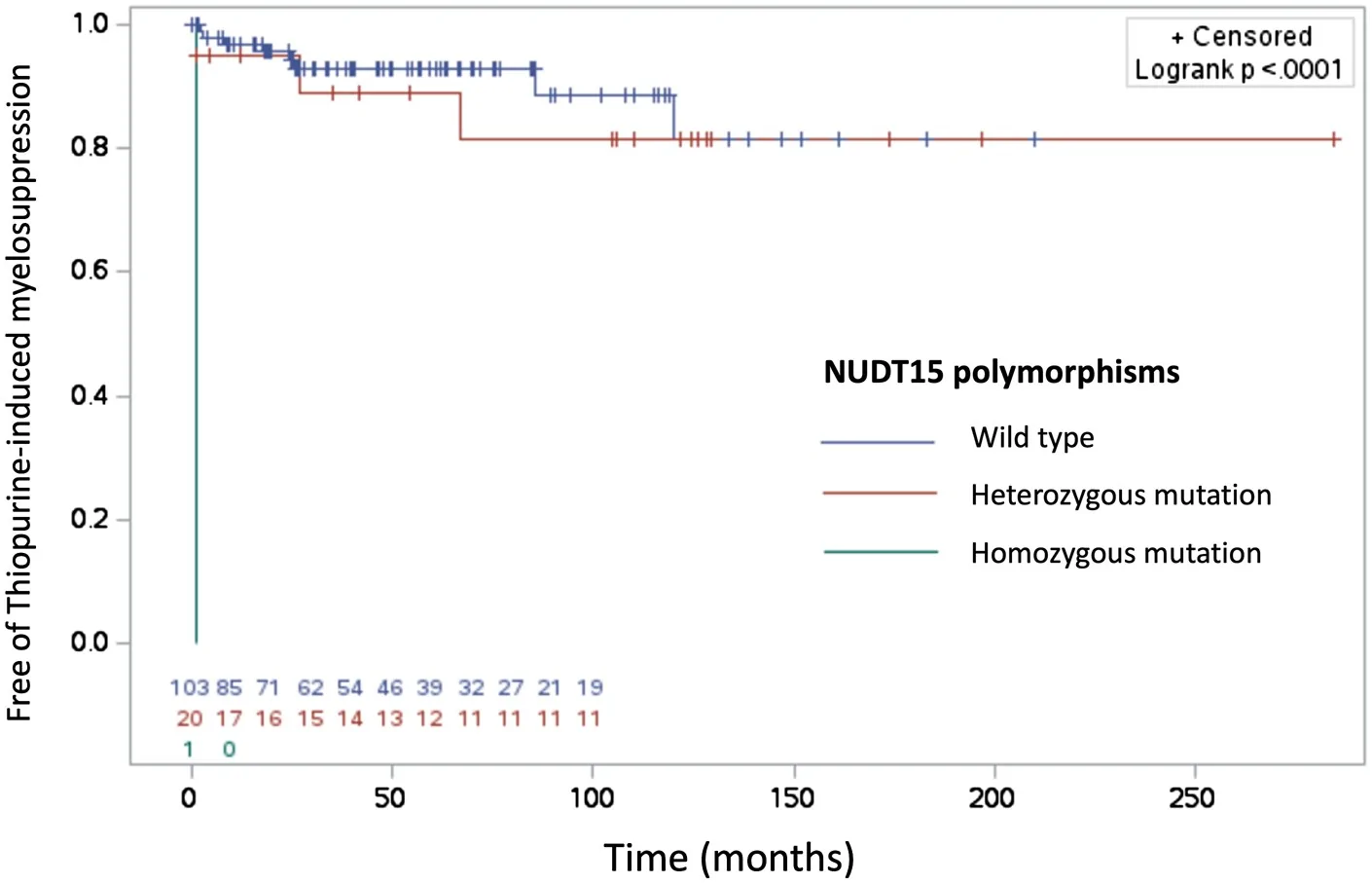
Kaplan-Meier survival curve showing the cumulative incidence of thiopurine-induced myelotoxicity (TIM) in participants stratified by NUDT15 polymorphism status.

Among the participants who developed TIM, 4 participants underwent dose reduction, while 8 discontinued thiopurine therapy. Nine patients were able to tolerate or restart thiopurines at lower doses, although recurrent TIM was observed in 6 of them. No deaths were reported in this study.

## Discussion

This study evaluated the prevalence of TPMT and NUDT15 polymorphisms and their association with TIM in 124 Thai IBD patients treated with thiopurines. TPMT polymorphisms were detected in 4.84% of participants, while NUDT15 polymorphisms were identified in 16.94%. TIM occurred in 12 participants (9.68%), with higher rates observed in those with NUDT15 polymorphisms (19.0%). TPMT polymorphisms were rare in our cohort with less common TIM (16.7%). Notably, 7 of 12 TIM cases occurred in participants without TPMT or NUDT15 polymorphisms. Most patients tolerated thiopurine dose adjustments, with no deaths reported.

Despite concerns regarding side effects, thiopurines, particularly azathioprine, remain among the most commonly prescribed medications for maintaining remission in IBD patients in resource-limited regions. Adverse effects of thiopurine therapy can arise as early as the first month, leading to treatment discontinuation in 19%-28% of cases.^18^^,^^19^ TIM is a dose-dependent adverse reaction influenced by genetic susceptibility, characterized by bone marrow suppression, resulting in anemia, leukopenia, or thrombocytopenia. It is one of the most common reasons for discontinuing thiopurine therapy. The TPMT gene, well-known for its association with myelosuppression, displays genetic variability, leading to significant interindividual differences in clinical efficacy and toxicity profiles during thiopurine treatment.^20^ The enzymatic activity of TPMT varies across populations, with high activity observed in 89% of individuals, intermediate activity in 11%, and low or absent activity in 0.3%.^11^ TPMT*2, *3A, *3B, and *3C account for over 95% of mutations and are linked to varying degrees of reduced enzyme activity. Among these, TPMT*3A is the most common variant (3%-5%) in Caucasians, leading to an approximate 25% reduction in enzyme activity compared to the wild-type allele.^21^

While the incidence of leukopenia ranges from 2% to 16% in Western populations,^22^^,^^23^ studies from Asia reported higher incidences of 20.6%-41.3%.^24–26^ However, the prevalence of TPMT mutations appears to be lower in Asians, and their effect on leukopenia is varied. A study involving 286 Korean IBD patients reported that 41.3% developed leukopenia. In this study, TPMT*1/*3C was detected in only 7 cases (2.4%), of which 5 cases were associated with leukopenia. Cox regression analysis demonstrated that the heterozygous *3C TPMT genotype was a significant risk factor for developing leukopenia compared to those with wild-type genotype (*P *= .02).^24^ Another retrospective study involving 253 patients with CD in China reported that 25.7% developed leukopenia, but they identified TPMT*3C in only 8 cases (3.2%). Of 8 cases, only 3 individuals with TPMT*3C experienced leukopenia, and no significant correlation was observed between TPMT genotype and the incidence of TIM.^26^ A systematic review and meta-analysis of 26 studies in South Asia reported a pooled prevalence of TPMT polymorphisms at 4.57%; among patients experiencing adverse effects, the pooled prevalence of TPMT polymorphisms was only 9.47%.^27^ These findings align with our results, demonstrating a frequency of TPMT polymorphisms of 4.8% without a significant association between TPMT polymorphisms and TIM observed. Therefore, TIM in Asians is likely caused by other mechanisms.

NUDT15 polymorphism has been reported to be associated with thiopurine-induced early leukopenia, particularly in Asian populations.^10^^,^^27–29^ This occurs due to the accumulation of cytotoxic TGTP metabolites, which are incorporated into DNA and trigger apoptosis. A study from China involving 732 IBD patients found that 177 (24.3%) developed leukopenia. Strong associations were observed between thiopurine-induced leukopenia and the NUDT15 variants c.415C > T, c.36_37insGGAGTC, and c.52G > A (*P *= 1.81 × 10^−20^, *P *= 4.74 × 10^−8^, and *P *= .04, respectively), while no association was found with TPMT genotypes (*P *= .25).^30^ A large prospective study of 1014 IBD patients from India also identified NUDT15 genotype as an independent risk factor for leucopenia, with hazard ratios of 11.31 for C/T, 31.28 for T/T, compared to the C/C diplotype.^31^ The meta-analysis in South Asia reported a pooled prevalence of NUDT15 mutations of 16.5%, higher than that of TPMT mutations of 4.57%. Among patients experiencing adverse effects, the pooled prevalence of NUDT15 was also significantly higher than TPMT mutations (49.51% vs. 9.47%).^27^ Recently, a systematic review and meta-analysis from 20 studies comprising 5232 IBD patients on the prevalence of NUDT15 variants and the incidence of TIM found that the C/T (intermediate metabolizer) diplotype is the most common NUDT15 polymorphism, with a pooled prevalence of 13%, followed by the T/T diplotype (poor metabolizer) at 2%. The prevalence of the C/T diplotype was highest in Japanese (20%) and Chinese (18%) populations. The incidence of early leukopenia was 20% in heterozygous carriers and 99% in patients with the homozygous mutation.^12^ Few studies have been conducted in Southeast Asian IBD populations.^32^^,^^33^ The Malaysian study examined the link between NUDT15 polymorphisms and TIM in a multiethnic IBD population.^33^ Among 102 patients on thiopurine therapy, 18.6% developed leukopenia. The NUDT15 c.415C > T variant was the most prevalent, detected in 57.9% of leukopenia cases, resulting in a shorter onset of leukopenia, while the c.52G > A variant was less identified in 10.5% of cases. The findings of our study align with previous research, identifying a similar rate of TIM, with NUDT15 polymorphisms present in 4/12 (33.3%). However, less common variants were not assessed.

Among the 12 cases of TIM, 7 occurred even at standard doses, without detectable major TPMT or NUDT15 polymorphisms. This suggests the involvement of unassessed minor variants or other genetic polymorphisms influencing TIM. Additionally, co-administration of systemic 5-ASA may play a role. Evidence indicates that 5-ASA can inhibit TPMT activity and interact with thiopurines, leading to elevated 6-TGN levels in 82%-100% of patients, thereby increasing leukopenia risk. However, this association requires further validation in more extensive studies.^34^^,^^35^

While NUDT15 genotyping is not yet included in international IBD guidelines, this should be considered in Southeast Asia due to its wide use. The Clinical Pharmacogenetics Implementation Consortium recommends genotype-guided thiopurine dosing across diseases and encourages NUDT15 screening in Asian populations due to its high prevalence in the region.^36^ A meta-analysis on the role of pharmacogenomics in the efficacy and safety of thiopurines in IBD found that pretreatment genotyping was associated with a reduction in hematologic adverse events (relative risk; RR = 0.71). NUDT15 genotyping significantly reduced these events (RR = 0.63), whereas TPMT genotyping showed no significant effect (RR = 0.97).^37^

To our knowledge, this is the first study to comprehensively evaluate both TPMT and NUDT15 polymorphisms and their association with TIM in Thai IBD patients. Although pharmacogenetic studies have previously been conducted in Thailand, these have mainly involved pediatric patients with acute lymphoblastic leukemia. Our findings therefore provide important disease-specific data for Thai IBD patients and contribute to the limited evidence currently available from Southeast Asia. However, there are some limitations. First, the relatively small sample size and the low frequency of TPMT variant carriers limited the statistical power to detect associations between TPMT polymorphisms and TIM. Notably, the TIM rate in our study was numerically higher among heterozygous TPMT carriers than among wild-type participants, although the difference was not statistically significant. This finding should therefore be interpreted with caution. Second, as a single-center, retrospective study, it may not fully represent the broader population, though it captures comprehensive data from all patients in the clinic. Third, uncommon mutations were not analyzed, limiting the ability to fully explain cases of TIM in patients without common genetic mutations. Lastly, thiopurine metabolites were not assessed, which may limit insights into enzyme activity related to TPMT polymorphisms. Further multi-center studies and prospective trials are needed to validate and expand upon these findings.

## Conclusion

NUDT15 polymorphisms are more common than TPMT variants in this study and are significantly associated with TIM. The NUDT15 genotyping may help identify individuals at higher risk of myelotoxicity, the decision to implement routine screening should be weighed against local cost-effectiveness and availability.

## Data Availability

Data, analytic methods, and study materials are available to other researchers upon request.
